# A homozygous mutation in the stem II domain of *RNU4ATAC* causes typical Roifman syndrome

**DOI:** 10.1038/s41525-017-0024-5

**Published:** 2017-07-10

**Authors:** Yael Dinur Schejter, Adi Ovadia, Roumiana Alexandrova, Bhooma Thiruvahindrapuram, Sergio L. Pereira, David E. Manson, Ajoy Vincent, Daniele Merico, Chaim M. Roifman

**Affiliations:** 1 Division of Immunology and Allergy, Department of Pediatrics, The Hospital for Sick Children and the University of Toronto, Toronto, ON Canada; 2 The Canadian Centre for Primary Immunodeficiency and The Jeffrey Modell Research Laboratory for the Diagnosis of Primary Immunodeficiency, The Hospital for Sick Children, Toronto, ON Canada; 3 The Centre for Applied Genomics, Genetics and Genome Biology, Peter Gilgan Centre for Research and Learning, The Hospital for Sick Children, Toronto, ON Canada; 4 Department of Diagnostic Imaging, The Hospital for Sick Children and the University of Toronto, Toronto, ON Canada; 5 The Department of Ophthalmology and Vision Sciences, The Hospital for Sick Children and the University of Toronto, Toronto, ON Canada; 6 Deep Genomics Inc., Toronto, ON Canada

## Abstract

Roifman syndrome (OMIM# 616651) is a complex syndrome encompassing skeletal dysplasia, immunodeficiency, retinal dystrophy and developmental delay, and is caused by compound heterozygous mutations involving the Stem II region and one of the other domains of the *RNU4ATAC* gene. This small nuclear RNA gene is essential for minor intron splicing. The Canadian Centre for Primary Immunodeficiency Registry and Repository were used to derive patient information as well as tissues. Utilising RNA sequencing methodologies, we analysed samples from patients with Roifman syndrome and assessed intron retention. We demonstrate that a homozygous mutation in Stem II is sufficient to cause the full spectrum of features associated with typical Roifman syndrome. Further, we demonstrate the same pattern of aberration in minor intron retention as found in cases with compound heterozygous mutations.

## Introduction

Roifman syndrome (OMIM# 616651) was first identified as a novel association of immunodeficiency, spondyloepiphyseal dysplasia, developmental delay, retinal dystrophy and unique facial dysmorphic features.^1, 2^ Additional features, such as autoimmune hepatitis, cytopenia, arthritis and renal tubular dysfunction^3–6^ have been less consistent (Table 1). While all patients described so far were reported to have humoral immunodeficiency, T cell abnormalities appear more common than previously appreciated.^7^

**Table 1:** Patients’ clinical characteristics

Roifman syndrome feature	Patient 2 and previous cases (*n* = 11)	Patient 1
Growth deficiency		
Prenatal	9/11	Yes
Postnatal	10/11	Yes
Neurologic manifestations		
Developmental delay	8/11	Yes
Hypotonia	8/11	Yes
Dysmorphic features		
Microcephaly	9/11	Yes
Narrow upturned nose	10/11	Yes
Long philtrum	11/11	No
Thin upper lip	11/11	No
Brachydactyly	10/11	Yes
Clinodactyly fifth finger	6/11	Yes
Sensineural hearing loss	1/11	No
Skeletal anomaly		
Epiphyseal dysplasia	11/11	Yes
Ophthalmologic changes		
Retinal dystrophy	5/11	Yes
Cardiac manifestations		
Cardiac non-compaction	1/11	No
VSD	1/11	No
Endocrine dysfunction		
Hypogonadotrophic hypogonadism	1/11	No
Renal disease	1/11	No
Hepatosplenomegaly	6/11	No
Immune aberrations		
Elevated eosinophils	3/11	No
Low IgG	6/11	Yes
Low antibody titres	11/11	Yes
Abnormal T cells	4/11	Yes
Atopy	6/11	Yes
Autoimmune features	5/11	No

**Fig. 1:**
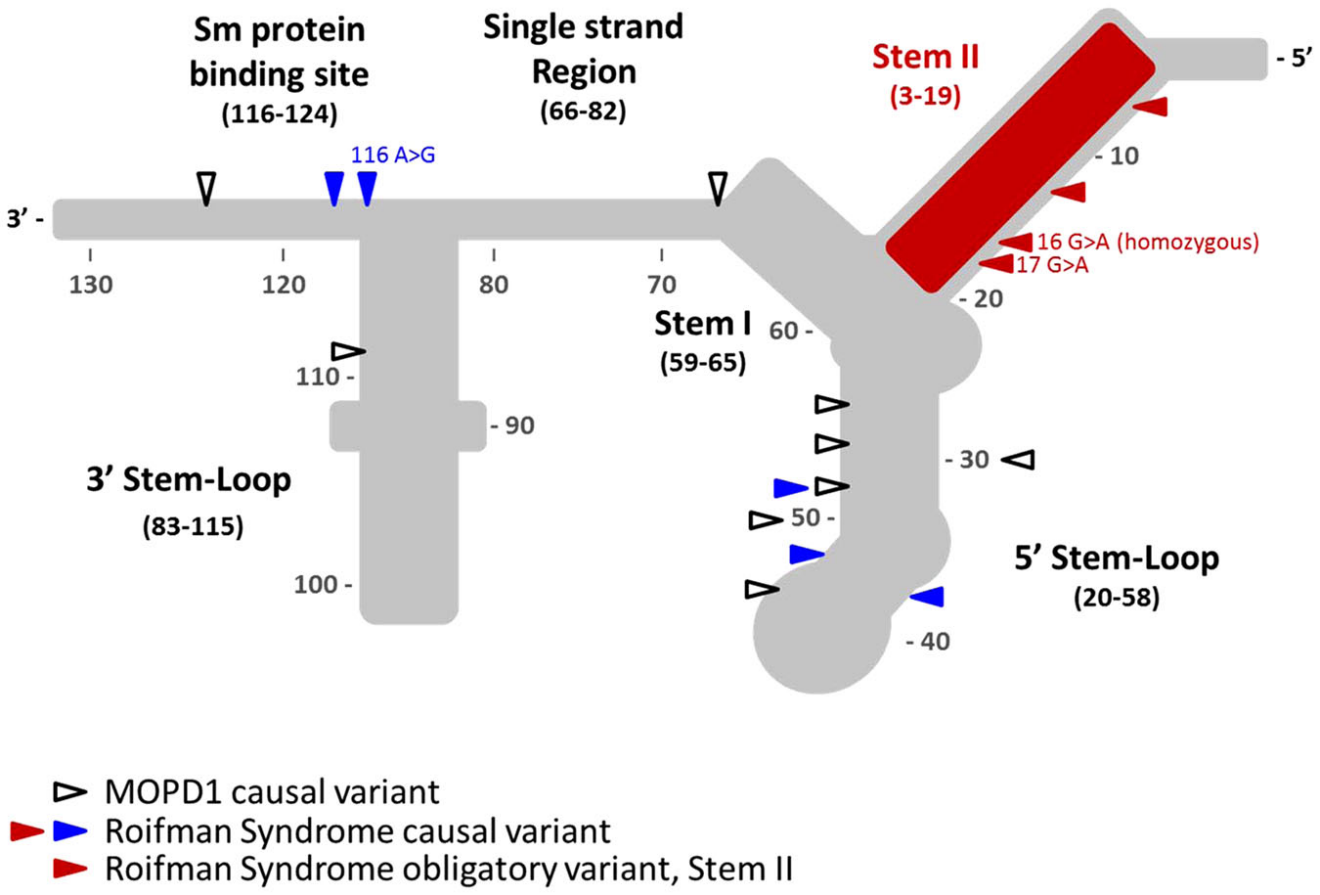
*RNU4ATAC* structural elements, and MOPD1 and Roifman syndrome causal variants. Stem I and Stem II are both elements at the 3′ and 5′ of U4atac, respectively, which base pair with U6atac. These elements are separated by an intramolecular 5′ stem-loop. Another stem loop is located at the 3′ end of U4atac. The Sm protein binding site is important for binding of the Sm proteins, which are important for the assembly of the complex and its import into the nucleus. The Stem II, Stem I, 5′ stem-loop and Sm binding site are all highly conserved. Delineated in *red* is the Stem II domain, which is obligatory for the pathogenesis of Roifman syndrome. Adapted from refs. 9, 10

Recently, compound heterozygote mutations in the *RNU4ATAC* gene were found to be the culprit for this disorder.^8^ This gene encodes for U4atac small nuclear RNA (snRNA), an essential component of the minor spliceosome, which is crucial for the correct splicing of about 800 genes carrying minor introns. The structural elements of the U4atac snRNA (Fig. 1) include two elements named Stem I and Stem II, which base pair the U6atac, required to form the catalytically active minor spliceosome. Stem I and Stem II are separated by a 5′ stem-loop. Another stem-loop, the 3′ stem loop, is followed by a sequence acting as a binding site for the Sm proteins, required for the assembly of the complex and its import into the nucleus. Roifman syndrome casual variants reported so far^8–10^ present a characteristic compound heterozygosity pattern, with one variant involving the 5′ stem-loop or the Sm protein-binding site, whereas the other variant which appears obligatory involves the Stem II element, a newly implicated and highly conserved element of the gene.

*RNU4ATAC* homozygous or compound heterozygous variants limited to Stem I, Sm protein binding site, and the 3′ stem-loop have been previously found in microcephalic osteodyslastic primordial dwarfism type I (MOPD1). MOPD1 is clinically distinct from Roifman syndrome, typically presenting early in life with a high pre-natal and post-natal lethality, major structural brain malformations, neuroendocrine dysfunction, very short and bowed limbs as well as dysmorphic features including proptotic eyes, prominent nose and micrognathia. Recently, adult cases with MOPD1 have been described.^11–15^ While clinical manifestations are far milder than typical MOPD1, they are still distinct from Roifman syndrome features, prominently lacking epiphyseal dysplasia and immunodeficiency.

We demonstrate here that mutations in Stem II region of *RNU4ATAC* are sufficient to inflict the full clinical features of Roifman syndrome, as demonstrated by a novel homozygous mutation in Stem II.

## Results

### Patient clinical characteristics

Patient 1 is the daughter of healthy consanguineous parents of Pakistani origin. She is the third of four siblings and there is no history of known immunodeficiency in her family. She was born at term with a low weight for gestational age of 1.4 kg. She then presented at the age of 11 months with recurrent pneumonias and ear infections, requiring multiple hospital admission for antibiotic treatment.

**Fig. 2:**
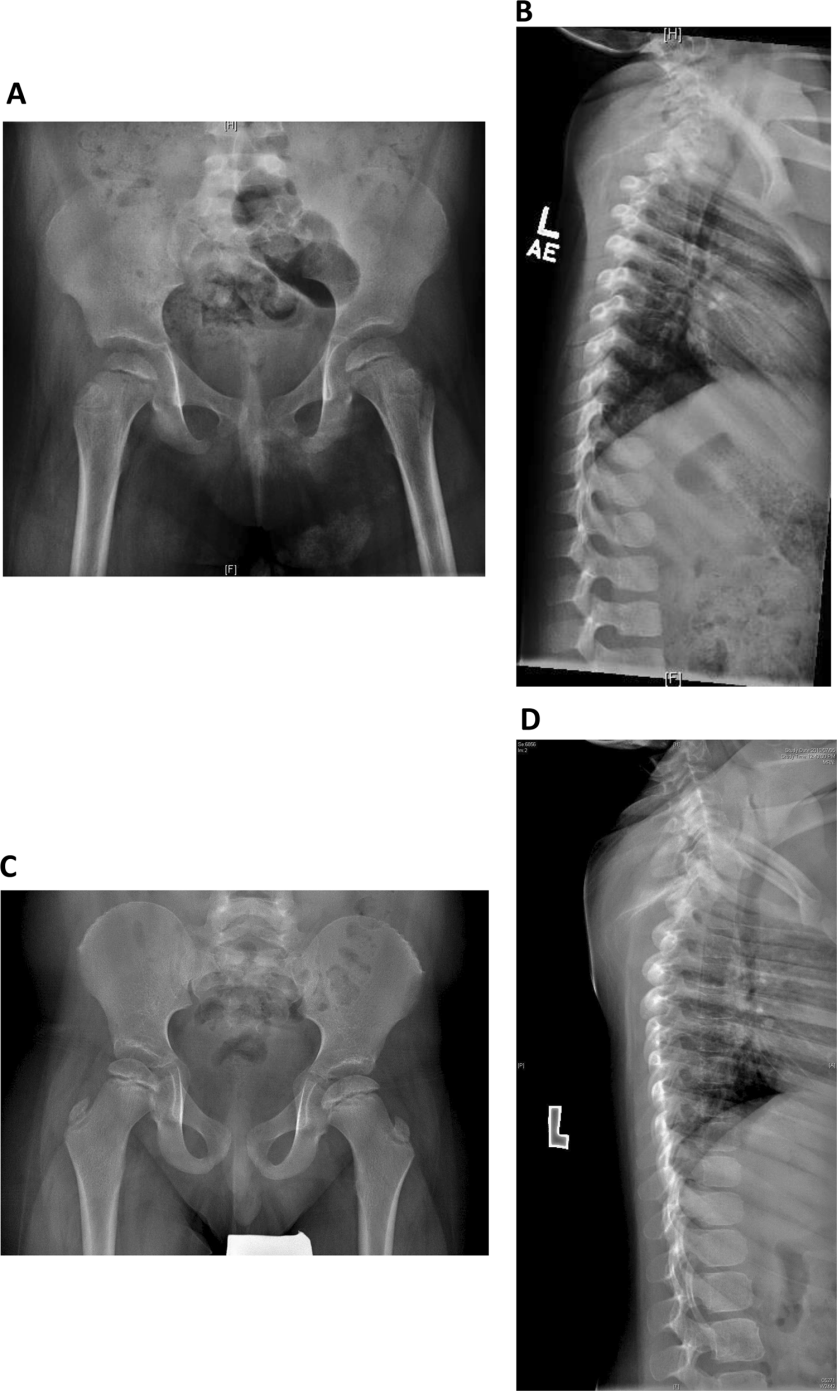
Skeletal features of Roifman syndrome in patients 1 and 2. **a** Pelvic X-ray of patient 1 featuring flattening of the humoral heads and shortening of the femoral necks, representing early stages of spondyloepiphyseal skeletal dysplasia. **b** Lateral spine X-ray of patient 1 featuring anterior vertebral notching of the lower thoracic vertebrae, and loss of lumbar lordosis. **c** Pelvic X-ray of patient 2 featuring bilateral small, flattened and slightly broadened femoral heads. **d** Lateral spine X-ray of patient 2 featuring loss of lumbar lordosis.

**Fig. 3:**
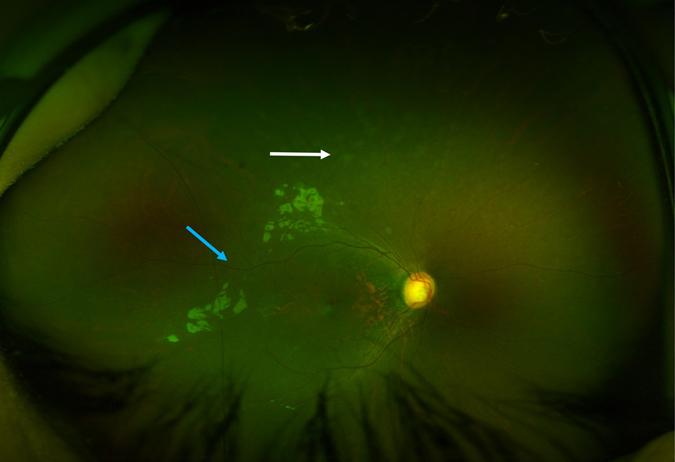
Retinal features of Roifman syndrome in patient 1. Retinal exam featuring normal optic discs, dull foveal reflex, attenuated retinal vessels (*blue arrow*) and background retinal pigment epithelial changes in the middle and far periphery (*white arrow*).

In addition to her infectious history, the patient had significant failure to thrive at presentation, along with significant atopy, presenting as asthma and eczema. Her weight subsequently improved with dietary management, but her height remained well below the third percentile for age. Skeletal survey revealed bilateral clinodactyly of the fifth fingers and spondyloepiphyseal dysplasia (Fig. 2a, b). She was noted to be microcephalic with head circumference of 40.8 cm at 6 months of age (between the third and tenth percentile for age), and to have dysmorphic features including upturned nares, low anterior hairline, prominent forehead and brachydactyly. She also had developmental delay: she sat at the age of 12 months, walked at the age of 2 years and started speaking at the age of 2.5 years. A formal developmental assessment was conducted at the age of 21 months, using the 20 Months Ages and Stages Questionnaire. She was found to have delays in both gross and fine motor skills. Language skills were assessed using the Communications and Symbolic Behavior Scales Developmental Profile. She was found to have age appropriate receptive language skills, but delayed expressive language skills. At the age of 6 years, she attends a regular class and requires the assistance of speech and occupational therapies. Ophthalmic evaluation was conducted due to symptoms of night blindness and revealed moderate–severe rod-cone dystrophy (Fig. 3). The patient’s clinical features, as compared to previous reports, are described in Table 1.

Immune work up (Table 2) revealed a relative paucity of CD8 + cells, and poor in vitro response to antigens. CD19 + B cell counts were low and she developed hypogammaglobulinemia with absent responses to vaccinations with tetanus, measles, mumps and varicella. Even after the age of 2 years, she was unable to mount a response to the polysaccharide pneumococcal vaccine.

**Table 2:** Patient 1–immune work up

	Patient 1	Normal values
Lymphocyte markers (cells/µl):		
CD3	3911	1600–6700
CD4	3503	1000–4600
CD8	436	400–2100
CD4:CD8 ratio	8	1.3–3.9
CD19	450	600–2700
CD3^-^CD56/CD16	356	200–1200
Immunoglobulins (g/l):		
IgG	2.4	4.5–14.3
IgA	0.2	0.2–1.0
IgM	0.4	0.2–1.8
IgE (IU/ml)	<25	<60
Specific antibodies:		
Anti-tetanus (IU/ml)	0.02	
Anti-Pneumococcus	3	>4
(fold increase)		
Anti-measles IgG	Negative	
Anti-mumps	Negative	
Anti-rubella	Positive	
Anti-varicella	Negative	
Antigen response:		
Candida	Negative	>20

The patient was started on intravenous immunoglobulin (IVIG) therapy and is tolerating her treatment very well, with dramatic improvement in reducing the frequency of infections.

**Fig. 4:**
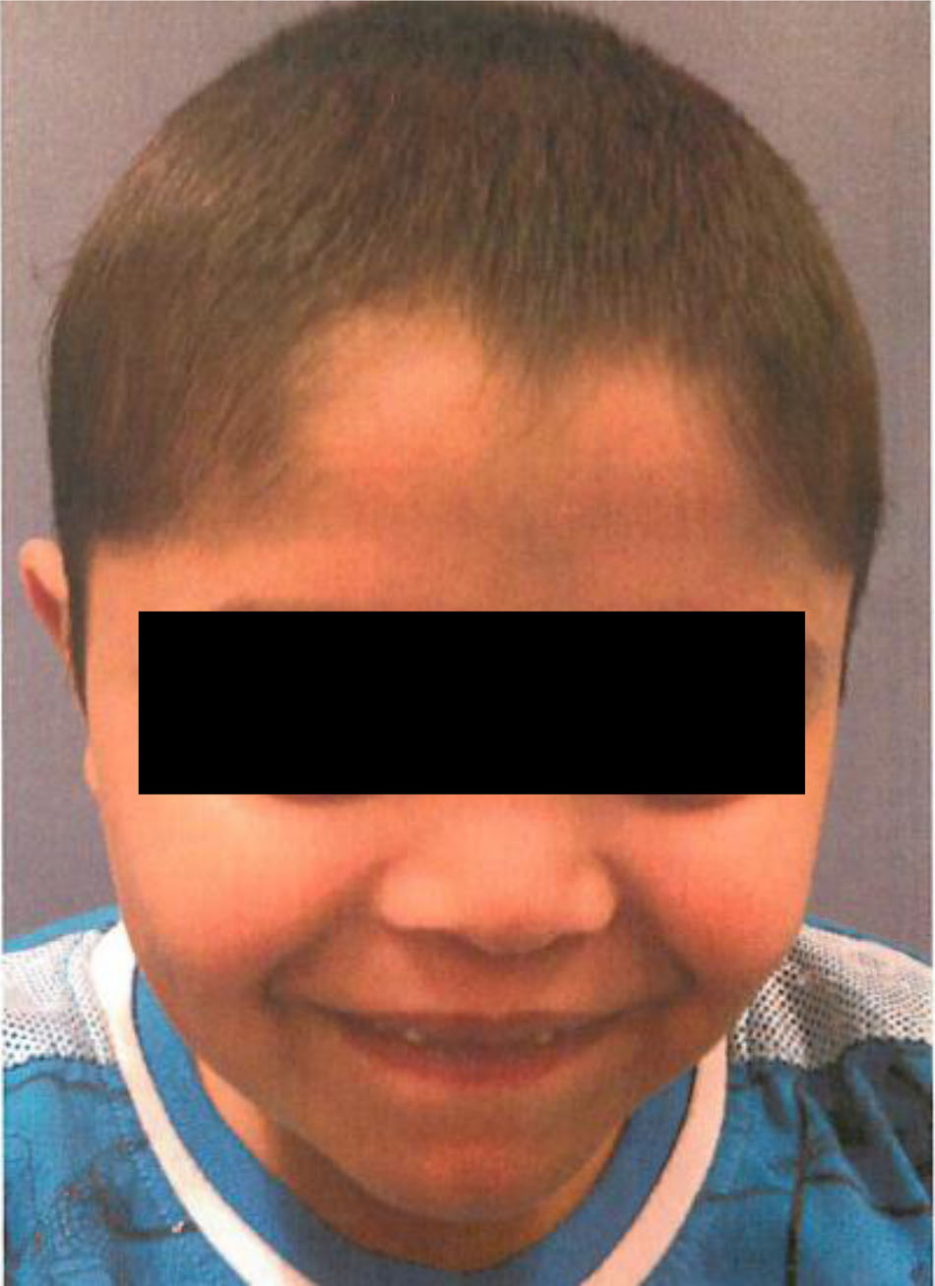
Facial features of the patient 2. Narrow palpebral fissures, a long philtrum and a thin upper lip are evident, in keeping with the classical facial features of Roifman syndrome patients. Informed consent was obtained for publication of patient images.

Patient 2 was reported previously.^9^ Briefly, he is a 12-year old-boy of Tamil descent, born at term and was small for gestational age. He first presented at 11 weeks of age with severe episodes of bilateral pneumonia and continued to suffer from recurrent pneumonia and asthma thereafter. Upon assessment at the age of 5 months, his head circumference was 39.5 cm (below the third percentile for age). Weight was at the tenth percentile for age and length was below the third percentile for age. Developmental assessments, performed at 11 months and 5 years of age, indicated delays in expressive language, gross and fine motor skills, while social skills were appropriate for age. He was found to have characteristic dysmorphic features (Fig. 4), spondyloepiphyseal dysplasia (Fig. 2c, d), as well as retinal dystrophy. A brain MRI revealed mild ventriculomegaly and prominent extra-axial cerebrospinal fluid spaces. An immunologic evaluation was significant for CD19 lymphopenia and hypogammaglobulinemia. He was unable to mount a long-standing response to tetanus and pneumococcal vaccines, and had non-reactive titres for measles, mumps, rubella and varicella, despite appropriate vaccination. He was therefore started on IVIG treatment at the age of 11 years. Similar to patient 1, the responses to T cells antigens were absent.

### Sanger sequencing

**Fig. 5:**
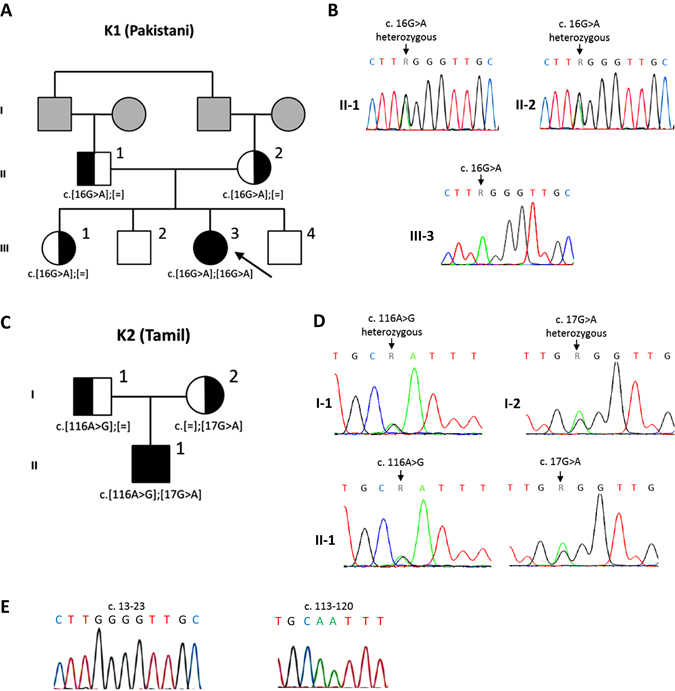
Pedigree of Roifman syndrome patients. **a** Pedigree of kindred 1 (K1), the family of patient 1, showing a c.16 G > A homozygous mutation. **b** Sequencing analysis of patient 1 and her heterozygous carrier parents. **c** Pedigree of kindred 2 (K2), the family of patient 2, showing a compound heterozygous mutation c.116 A > G/17 G > A. **d** Sequencing analysis of patient 2 and his heterozygous carrier parents. **e** Sequencing analysis of wild type *RNU4ATAC*. *Circles* represent female subjects, while *squares* denote male subjects. The *black colour* represents an affected status. The *grey colour* represents individuals who were unavailable for sequencing. The *white colour* represents affected non carrier, and *half black–half white colour* represents non affected heterozygous carrier. [=] indicates no variant detected.

Sanger sequencing of the *RNU4ATAC* gene for Patient 1 revealed a c.16 G > A homozygous mutation. Both parents were found to be heterozygous carriers of the same mutation. An analysis of other family members revealed another sister who is a carrier of the same mutation and two non-carrier brothers (Fig. 5a, b). This variant was previously reported in a compound heterozygous patient^8^ and was absent in 800 samples of whole genome sequences obtained for unrelated conditions.

Sanger sequencing for patient 2 revealed a compound heterozygous mutation c.116 A > G/17 G > A of the *RNU4ATAC* gene. The mother was found to be a carrier of the 17 G > A variant and the father of the 116 A > G variant (Fig. 5c, 5d).

### RNA sequencing (RNA-Seq)

**Fig. 6:**
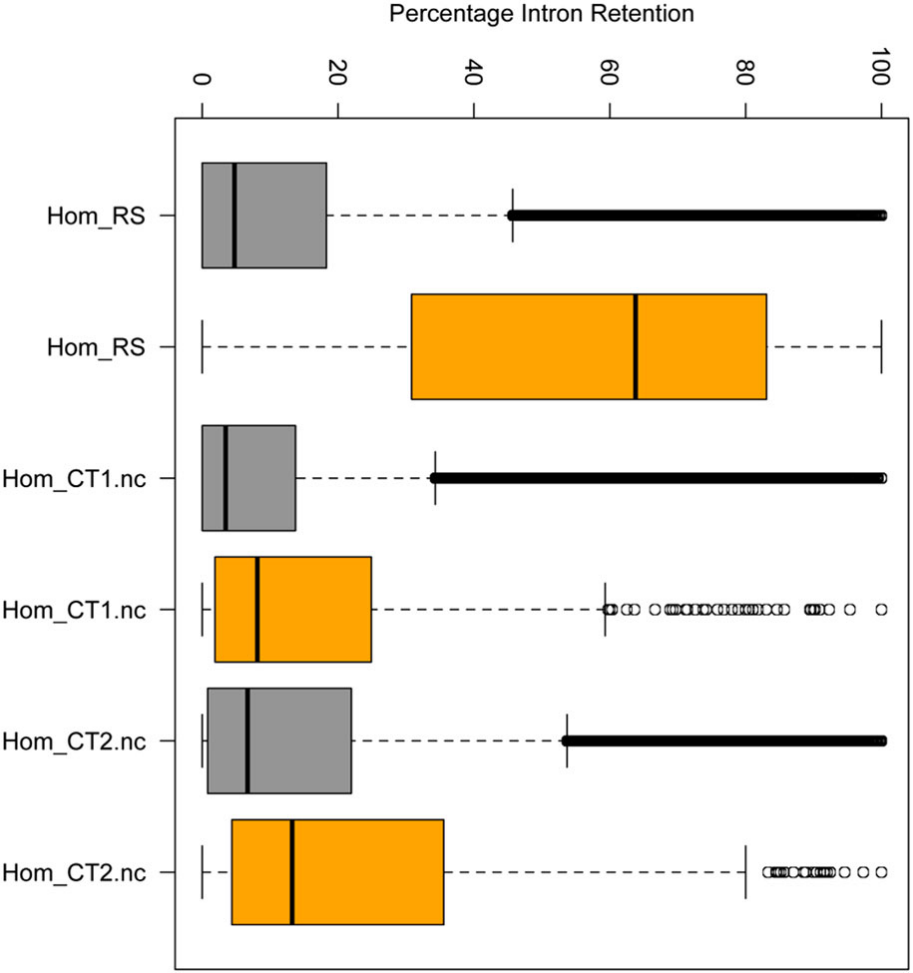
Percentage intron retention (PIR) in patient 1 and unaffected family members. PIR across different introns is displayed as a boxplot: the affected patient 1 clearly displays greater minor intron retention than the unaffected wild-type siblings, whereas major intron retention levels are lower and similar across samples. Hom_RS-patient 1; Hom_CT1.nc-unaffected, non-carrier male sibling of patient 1, aged 3 months; Hom_CT2.nc-unaffected, non-carrier male sibling of patient 1, aged 9 years. *Grey boxes*–major intron retention levels, *orange boxes*–minor intron retention levels.

**Fig. 7:**
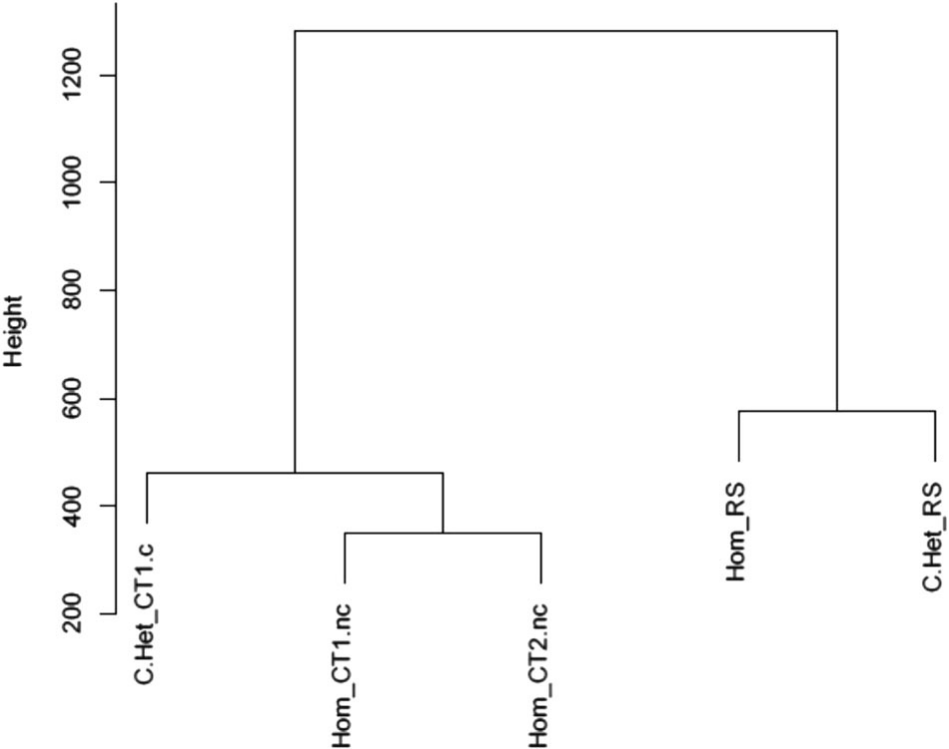
Cluster analysis for minor intron retention of affected subjects vs. controls. Cluster analysis was performed using the euclidean distance metrics. A clear discrimination of minor intron retention levels is demonstrated between affected subjects of both heterozygous and homozygous mutations, and their unaffected controls. C.Het_CT1.c-Unaffected carrier mother of patient 2; Hom_CT1.nc-unaffected, non-carrier male sibling of patient 1, aged 3 months; Hom_CT2.nc-unaffected, non-carrier male sibling of patient 1, aged 9 years; Hom_RS-Patient 1; C.Het_RS-Patient 2.

To confirm the presence of minor splicing intron retention, RNA-Seq was performed on peripheral blood cells from kindred 1 (the affected girl and her non-carrier brothers) and kindred 2 (the affected boy and his carrier father). Using the vast-tools analysis pipeline, consistent minor intron retention was detected in affected compared to unaffected subjects, with no significant differences in major intron retention (Fig. 6). A cluster analysis was performed to show that the minor intron retention levels perfectly discriminate affected subjects (with homozygous Stem II or compound heterozygous Roifman Syndrome mutations) from family-matched unaffected controls (Fig. 7); results are identical using two different distance metrics (Manhattan and Euclidean, see Fig. 7 and Supplementary Fig. 1).

The RNA-seq-aligned reads in kindred 1 confirmed the presence of the homozygous mutation in patient 1 (Supplementary Fig. 2). In addition, RNU4ATAC was expressed at much higher levels patient 1 compared to her unaffected, age-matched siblings (log2 ratio = 5.73). A similar pattern was observed for the other minor spliceosome snRNA RNU11 and RNU12 (log2 ratio = 5.77, 6.40) (Supplementary Table 1). This likely reflects a compensatory expression response, which is however unable to compensate for the minor spliceosome defect.

## Discussion

Since its first description in 1997 (see ref. 1), Roifman syndrome has been recognised in patients with different ethnic backgrounds.^1, 2, 4–6, 10^ More recently, the cause for this syndrome was elucidated as the non-coding *RNU4ATAC* gene on chromosome 2, which is a part of the minor spliceosome complex.^8^ All mutations found so far have been compound heterozygous mutations with one obligatory variant involving the Stem II domain.^8–10^ The Stem II element of the yeast U4 snRNA gene (SNR14), the conserved parallel of the human RNU4ATAC, was shown to be highly sensitive to mutagenesis, whereas the stem 5′ was relatively tolerant of mutations.^16^ In their work, Hu et al. implicated the Stem II domain as crucial for the interaction of the U4atac with U6atac,^16^ which is a necessary step in the assembly of the catalytically active complex.

In contrast with these findings, the more severe phenotype in humans is that of MOPD1, which is caused by compound heterozygous or homozygous mutations in the same gene, involving the 5′ stem loop, 3′ stem loop, Stem I or Sm protein binding site, but excluding the Stem II element (Fig. 1).^8^ The various mutations in MOPD1 present different functional implications^17^- while one mutation (124 G > A) resulted in reduction of RNU4ATAC levels, other mutations, namely the ones in the stem 5′ loop, impaired RNU4ATAC’s binding to NHP2L1 and/or PRPF31, both essential proteins for the assembly of the active minor spliceosome complex. Clinically, while most MOPD1 present with early lethality,^18, 19^ there have been several reports^11–15^ of older patients with a milder phenotype, yet still, features are completely different from the set of manifestations observed in Roifman syndrome.

Here we describe a homozygous case of Roifman syndrome, caused solely by the c.16 G > A mutation in Stem II. This confirms that a mutation in this element is sufficient to produce the full phenotype of Roifman syndrome, including skeletal dysplasia, retinal dystrophy, immunodeficiency and developmental delay. Our patient suffers from the full severity of clinical manifestations described previously in other subjects.^1, 2, 4–6, 8–10, 20^

To investigate whether the biological effect of this mutation is similar to previously described transcriptional aberration observed in cases with compound heterozygous mutations, we compared RNA-Seq analysis between these genotypes. We show that the percentage of minor intron retention across different introns in patient 1 is far greater than her unaffected wild-type siblings. This pattern confirms previous observations in Roifman syndrome cases who carry compound heterozygous mutations. To show parity with cases caused by compound heterozygous mutations, we have performed cluster analysis comparing minor intron retention in patients 1 and 2. We demonstrated clear discrimination of minor intron retention levels between affected subjects (both heterozygous and homozygous mutations) compared with unaffected controls.

Our findings prove a clear genotype–phenotype relationship with mutations in Stem II conferring a distinct milder phenotype, which nevertheless involves the immune as well as other systems. The pathogenesis of this differential expression is yet to be elucidated, however, it is clear, that variants in Stem II confer a differential loss of function of RNU4ATAC, implicating the genes affected.

Previous reports have highlighted the humoral defects in Roifman syndrome.^1, 2, 4, 5, 8–10, 21^ However, the common occurrence of atopy, as well as the high prevalence of autoimmune manifestations, such as immune cytopenia, autoimmune hepatitis and colitis, indicates T cell immune dysregulation or aberrant T cell function. Moreover, in addition to having multiple sino-pulmonary infections, these patients suffer severe viral and fungal infections which are consistent with a T cell defect.^5, 8, 9, 21^ Indeed, the inability of T cells to respond to antigens support that notion. Together, this redefines the immune phenotype of Roifman syndrome as a combined immunodeficiency.

In summary, we have shown here that biallelic mutations limited to Stem II of the *RNU4ATAC* are sufficient to produce all features of Roifman syndrome.

## Conclusion

We present two cases of a heterozygous and a homozygous mutation involving the Stem II element of *RNU4ATAC* thereby causing Roifman syndrome. These mutations highlight Stem II as the element within this gene, which is responsible for the manifestations of this syndrome.

## Methods

### Patients

Patient information was collected prospectively and retrospectively from medical records and entered to the Canadian Centre for Primary Immunodeficiency Registry (SickKids Research Ethics Board approved protocol no. 1000005598). Informed consent was obtained for the patients described.

### Serum concentration of immunoglobulin and specific antibodies

Serum concentrations of immunoglobulins were measured by nephelometry. Levels of serum antibodies to tetanus were measured by ELISA.

### T and B cell proliferative response

Lymphocyte proliferative responses to mitogens including phytohemagglutinin and anti-CD3 antibodies, and to a panel of recall antigens (including candida, tetanus, herpes zoster, and cytomegalovirus) were determined by thymidine incorporation. All assays were performed in triplicate and were compared with simultaneously stimulated normal controls.

### Sequencing analysis

Patient’s genomic DNA was extracted from peripheral blood lymphocytes using the Geneaid Genomic DNA Mini Kit. Genomic DNA was amplified by PCR with specific primers designed upstream and downstream of the *RNU4ATAC* gene. Sequencing was done using GenomeLab Dye Terminator Cycle Sequencing Quick Start Kit (Beckman Coulter) and analysed on CEQ 8000 Genetic Analysis System (Beckman Coulter).

### RNA sequencing

RNA library preparation and massively parallel sequencing was done for patient 1 and her unaffected mother and siblings as well as from patient 2 and his unaffected mother in The Centre for Applied Genomics, The Hospital for Sick Children. Quality of total RNA samples was checked on an Agilent Bioanalyzer 2100 RNA Nano chip following Agilent Technologies’ recommendation. Concentration was measured by Qubit RNA HS Assay on a Qubit fluorometer (ThermoFisher). RNA library preparation was performed following the NEB NEBNext Ultra Directional Library Preparation protocol using 1000 ng of total RNA as input material; total RNA was enriched for poly-A mRNA, fragmented into the 200–300-bases range for 4 min at 94 °C and converted to double stranded cDNA, end-repaired and adenylated at the 3′ to create an overhang A to allow for ligation of Illumina adaptors with an overhang T; library fragments were amplified under the following conditions: initial denaturation at 98 °C for 30 s, followed by ten cycles of 98 °C for 10 s, and 65 °C for 75 s, and finally an extension step for 5 min at 65 °C; at the amplification step, each sample was amplified with a different indexed adaptors to allow for multiplex sequencing. One microlitre of each RNA libraries was loaded on a Bioanalyzer 2100 DNA High Sensitivity chip (Agilent Technologies) to check for sizing and absence of primer dimers; RNA libraries were quantified by qPCR using the Kapa Library Quantification Illumina/ABI Prism Kit protocol (KAPA Biosystems) following the manufacturer’s recommended protocol. Libraries were pooled in equimolar quantities and paired-end sequenced on a High Throughput Run Mode flowcell with the V4 sequencing chemistry on an Illumina HiSeq 2500 platform following Illumina’s recommended protocol to generate paired-end reads of 126-bases in length.

Trim Galore^22^ v. 0.4.0 was used to trim the low-quality ends and adaptors from the RNA-seq reads. The raw trimmed reads were aligned to the reference genome hg19 using Tophat v. 2.0.11 (see ref. 23). Intron retention was analysed using vast-tools version 0.3.0 (see ref. 24). Clustering was performed using the Euclidean and Manhattan distance in R version 3.3.0 (ref. 25).

### Data availability statement

All data generated or analysed during this study are included in this published article and its supplementary information files.

## Electronic supplementary material

Supplementary Data

Supplementary Figure 1

Supplementary Figure 2

Supplementary Table 1
