# Supplementary material for: A homozygous mutation in the stem II domain of RNU4ATAC causes typical Roifman syndrome
Source: NPJ Genom Med. 2017 Jul 10;2:23. doi: 10.1038/s41525-017-0024-5 (PMC5677950; doi:10.1038/s41525-017-0024-5)
Supplement: Supplementary file 1 — Supplementary Data [file 41525_2017_24_MOESM1_ESM.docx]

**SUPPLEMENTARY DATA**

**Supplementary Figure 1:** **Cluster analysis for minor intron retention of affected subjects versus controls**. Cluster analysis was performed using the Manhattan distance metrics and showed similar results to the analysis done with euclidean distance metrics. A clear discrimination of minor intron retention levels is demonstrated between affected subjects of both heterozygous and homozygous mutations and their unaffected controls. C.Het_CT1.c-Unaffected carrier mother of patient 2; Hom_CT1.nc- Unaffected, non-carrier male sibling of patient 1, aged 3 months; Hom_CT2.nc- Unaffected, non-carrier male sibling of patient 1, aged 9 years; Hom_RS-Patient 1; C.Het_RS-Patient 2.

**Supplementary Figure 2: RNA-seq aligned reads in patient 1 versus carrier and non-carrier, unaffected family members.** The RNA seq aligned reads in RNU4ATAC confirms the presence of the homozygous mutation in the affected subject. The G>A substitution is observed in all reads for the homozygous G>A patient. Only one unaffected carrier shows the heterozygous substitution for part of its reads, but the number of reads spanning the position is small. The unaffected non-carriers do not show any substitution at this position. HetC1 – Unaffected, carrier female sibling of patient 1, aged 12 years, HetC2- Unaffected, carrier mother of patient 1, aged 43 years, Hom_RS -Patient 1, NC1- Unaffected, non-carrier male sibling of patient 1, aged 3 months; NC2- Unaffected, non-carrier male sibling of patient 1, aged 9 years.

**Supplementary Table 1: Relative expression of minor spliceosome components in Patient 1 versus carrier and non-carrier family member controls.** Expression of three minor spliceosome components (RNU11, RNU12 and RNU4ATAC) is increased in Patient 1 compared to her non carrier siblings as well as compared to her carrier sister and mother. BH-FDR- Benjamini-Hochberg False Discovery Rate. CT- control. HetC1 – Unaffected, carrier female sibling of patient 1, aged 12 years, HetC2- Unaffected, carrier mother of patient 1, aged 43 years, Hom_RS -Patient 1, NC1- Unaffected, non-carrier male sibling of patient 1, aged 3 months; NC2- Unaffected, non-carrier male sibling of patient 1, aged 9 years. RPKM- Reads Per Kilobase per Million mapped reads. RS-Roifman syndrome.
