# Supplementary figures and images for: A homozygous mutation in the stem II domain of RNU4ATAC causes typical Roifman syndrome
Source: NPJ Genom Med. 2017 Jul 10;2:23. doi: 10.1038/s41525-017-0024-5 (PMC5677950; doi:10.1038/s41525-017-0024-5)

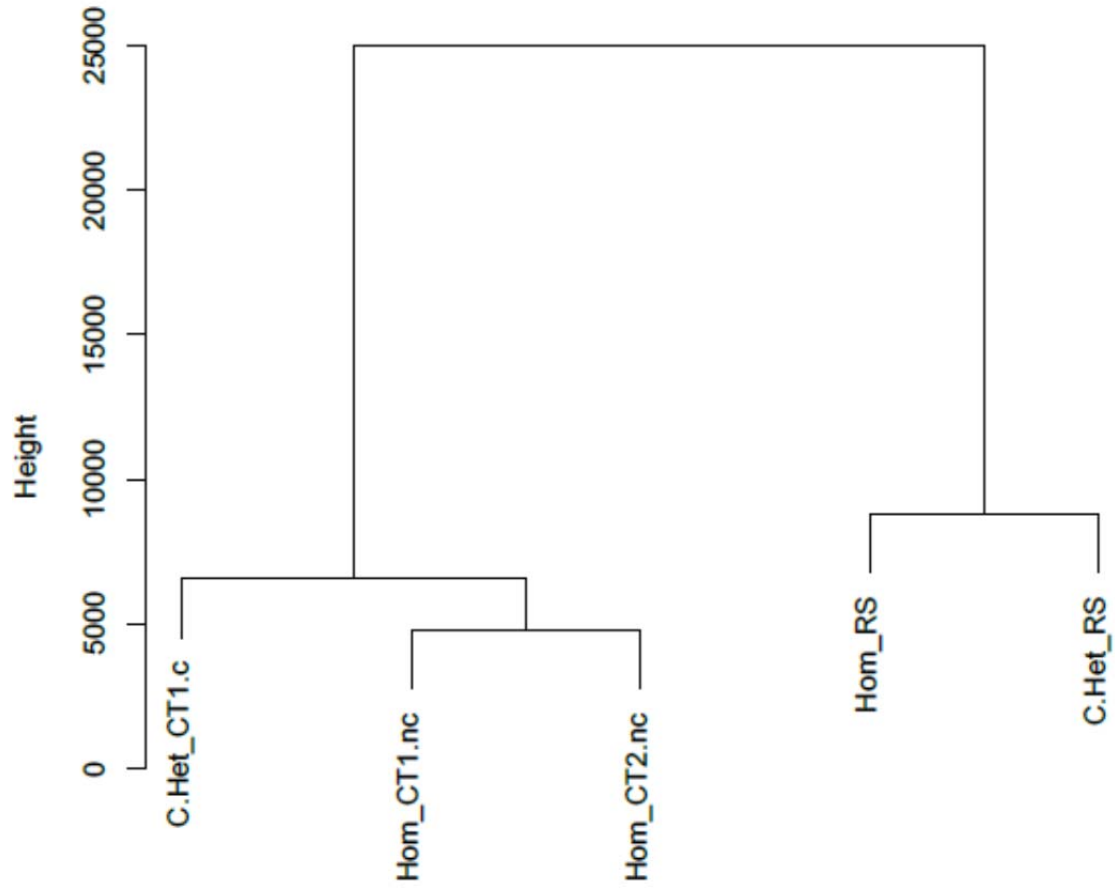

Supplement: Supplementary file 2 — Supplementary Figure 1 [file 41525_2017_24_MOESM2_ESM.pdf]

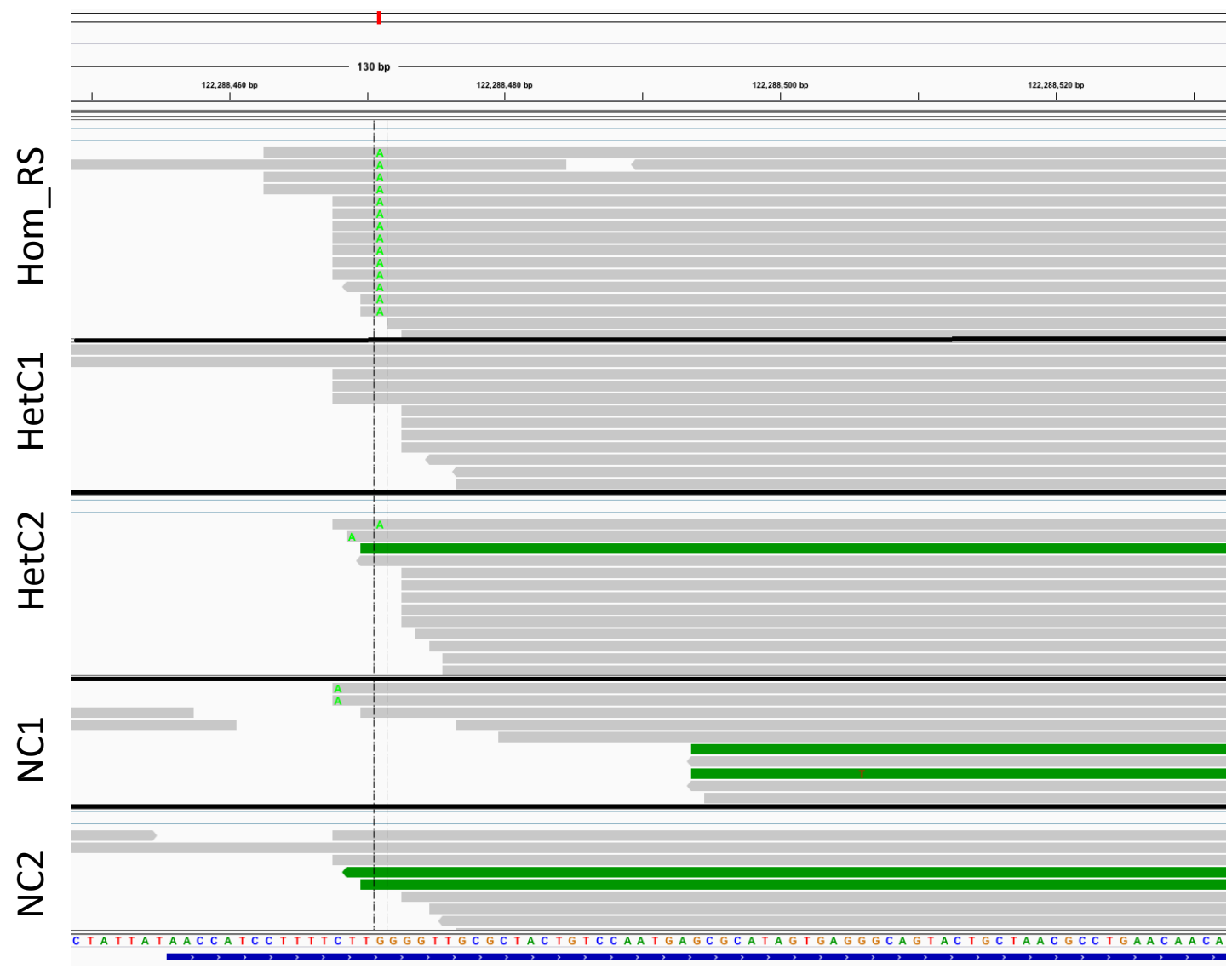

Supplement: Supplementary file 3 — Supplementary Figure 2 [file 41525_2017_24_MOESM3_ESM.pdf]
